# An anatomic anal sphincter-saving procedure for rectal cancers located at anorectal junction

**DOI:** 10.1186/s12957-019-1672-y

**Published:** 2019-08-02

**Authors:** Theodoros Mariolis-Sapsakos, Giannos Psathas, Taxiarchis Konstantinos Nikolouzakis, Konstantinos Laschos, Charikleia Triantopoulou, Gerasimos Bonatsos, John Tsiaoussis

**Affiliations:** 1 Consultant General Surgeon Surgical Department, Agioi Anargyroi General and Oncologic Hospital of Kifisia, National & Kapodistrian University of Athens, University of Athens, Athens, Greece; 2 Surgical Department, Agioi Anargyroi General and Oncologic Hospital of Kifisia, University of Athens, Athens, Greece; 3 Laboratory of Anatomy-Histology-Embryology, Medical School of Heraklion, University of Crete, Heraklion, Greece; 4 Oncology Pathology Department, Agioi Anargyroi General and Oncologic Hospital of Kifisia, Athens, Greece; 5 Radiology Department, Konstantopouleio General Hospital, Athens, Greece; 6 Surgical Department, Agioi Anargyroi General and Oncologic Hospital of Kifisia, National & Kapodistrian University of Athens, University of Athens, Athens, Greece

**Keywords:** Rectal cancer, Hemilevator excision, Anorectal function, Sphincter saving

## Abstract

**Background:**

This study aims to present the feasibility of the open approach of hemilevator excision (HLE) as a promising alternative of the laparoscopic and/or robotic ones for the treatment of low rectal cancer extending to the ipsilateral puborectalis muscle.

**Methods:**

A 60-year-old male patient with a high-grade differentiated rectal adenocarcinoma at the right side of the lower rectum invading puborectalis muscle. The proposed operation consists of a combination of extralevator abdomino-perineal excision (ELAPE), intersphicteric resection (ISR), and low anterior resection (LAR) since it resects the ipsilateral to tumor levator ani muscle (LAM) from its attachment at the internal obturator fascia and the deep part of ipsilateral external anal sphincter (EAS), while the distal part of dissection is completed in the intersphincteric space taking out the internal anal sphincter (IAS). At the contralateral side of the tumor, the dissection plane follows the classic route of LAR.

**Results:**

Pathology proved the oncologic adequacy of resection. MRI at the fourth postoperative week showed clearly the right aspect of anorectal junction free of tumor. Anorectal manometry revealed a fair anorectal function which is in accordance with the findings of clinical assessment of patient after restoring large bowel continuity (post-op Wexner score, 7).

**Conclusion:**

This is the first case of the open HLE that seems to be a good alternative compared to ELAPE or conventional APR, as it offers oncologic adequacy and a fair anorectal function.

## Background

The treatment of cancer of the rectal lower third has been a challenging issue over time. Back in 1908, Ernest Miles first described the abdomino-perineal excision (APE) [1]. Even after 110 years, this technique remains the standard choice for low rectal cancers (mainly found up to 5 cm from the anal verge) according to the ESMO guidelines for rectal cancer [2]. However, in terms of the oncologic outcome, it was found that this technique is not so effective [3]. In order to address this problem, a new technique was later described; the extralevator abdomino-perineal excision (ELAPE). ELAPE provides a cylindrical specimen in order to decrease the risk of involved circumferential resection margins (CRM) and to reduce the risk of intraoperative tumor perforation. However, both of them bare a major drawback: the patient ends up with a permanent colostomy. For this reason, another technique was proposed, the intersphicteric resection (ISR) [4]. This technique is applicable for the resection of low rectal tumors that do not invade the external sphincter and the oncologic results are indeed acceptable [5]. The open hemilevator excision (HLE) presented here is a promising approach for patients with a tumor of the lower rectum and an ipsilateral infiltration of puborectalis muscle with no distant metastases. These patients should have an efficient anorectal function based on manometric evaluation and clinical assessment with the Wexner scale score for incontinence [6].

## Methods

**Fig. 1:**
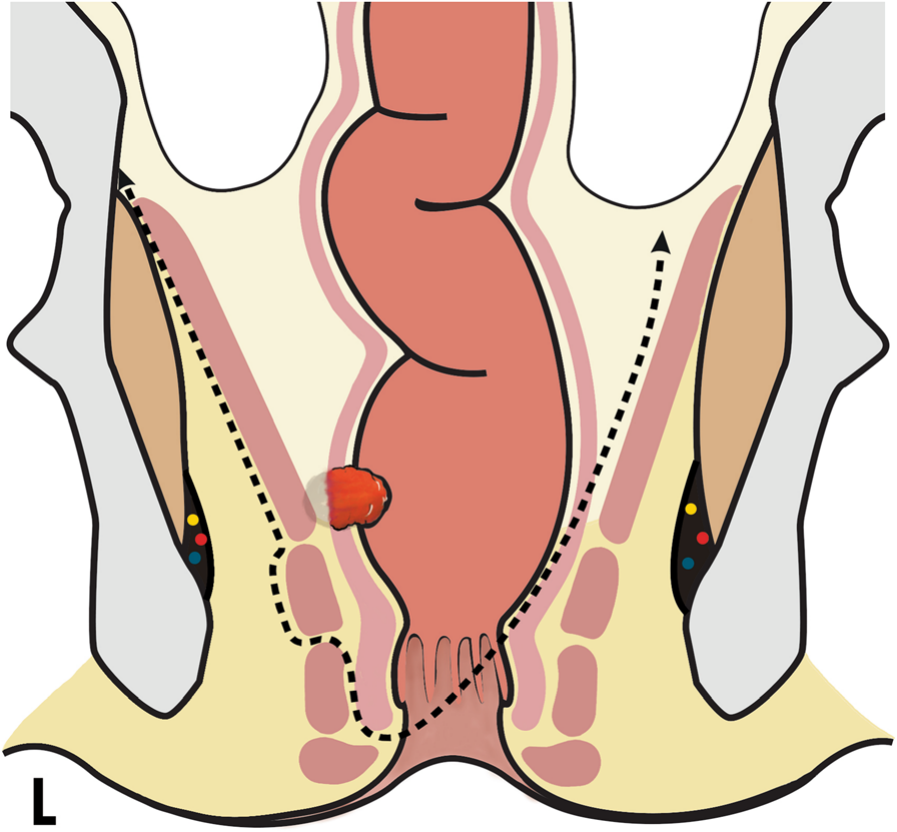
Coronal schematic representation of hemilevator excision and partial resection of the deep portion of ipsilateral external anal sphincter

**Fig. 2:**
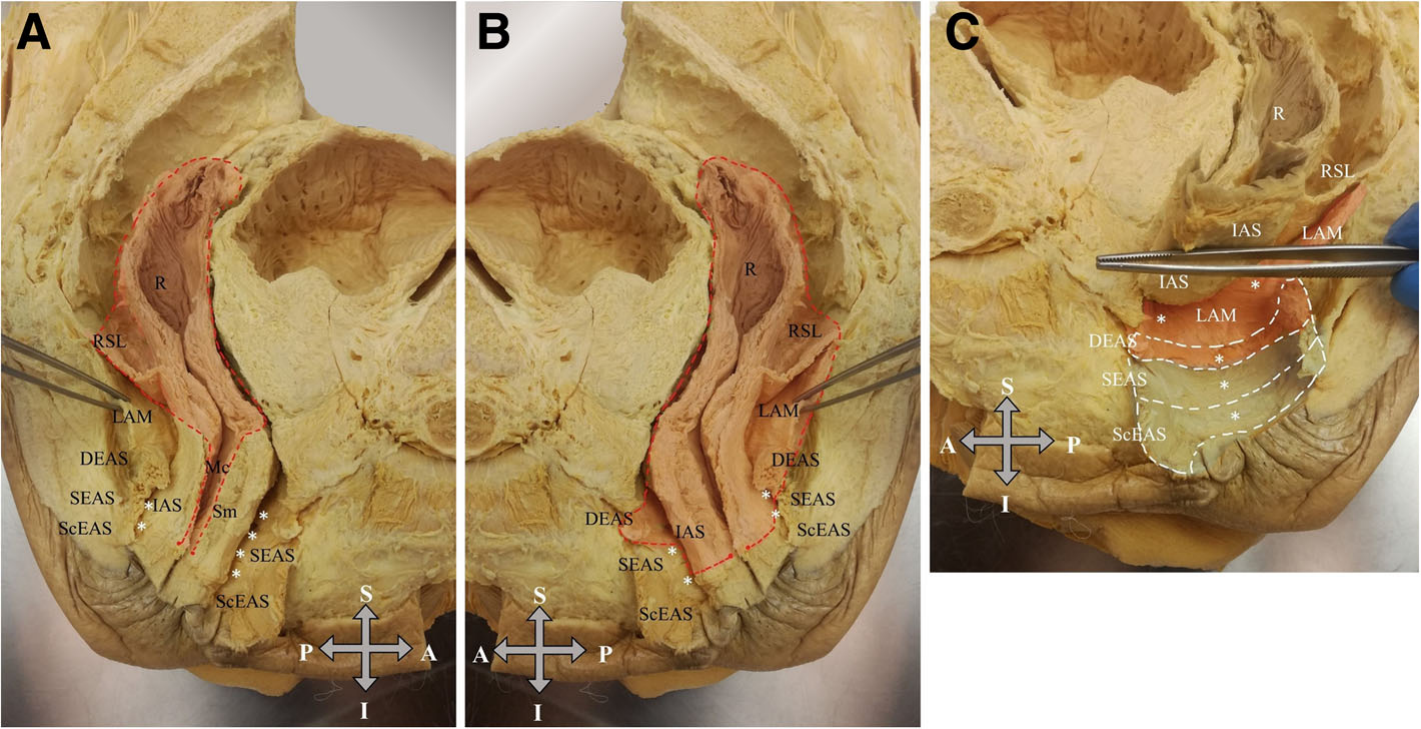
**a** Median sagittal plane in a male cadaveric left hemipelvis. S, superior; A, anterior; P, posterior; I, inferior; R, rectum; RSL, rectosacral ligament; LAM, levator ani muscle; DEAS, deep part of the external anal sphincter; SEAS, superficial part of the external anal sphincter; ScEAS, subcutaneous part of the external anal sphincter; IAS, internal anal sphincter; Mc, the rectal mucosa; Sm, rectal submucosa. The white asterisk represents the intersphincteric space. The dashed red line represents the surgical plane. The red shade represents the excised specimen. **b** Median sagittal plane in a male cadaveric right hemipelvis. S, superior; A, anterior; P, posterior; I, inferior; R, rectum; RSL, rectosacral ligament; LAM, levator ani muscle; DEAS, deep part of the external anal sphincter; SEAS, superficial part of the external anal sphincter; ScEAS, subcutaneous part of the external anal sphincter; IAS, the internal anal sphincter. The white asterisk represents the intersphincteric space. The dashed red line represents the surgical plane. The red shade represent the excised specimen. **c** Median sagittal plane in a male cadaveric right hemipelvis. S, superior; A, anterior; P, posterior; I, inferior; R, rectum; RSL, rectosacral ligament; LAM, levator ani muscle; DEAS, deep part of the external anal sphincter; SEAS, superficial part of the external anal sphincter; ScEAS, subcutaneous part of the external anal sphincter; IAS, internal anal sphincter. The white asterisk represents the intersphincteric space. The dashed white line represents external anal sphincter complex. The red shade indicates the excised part of the external anal sphincter and levator ani muscle and the blue shade indicates the part of the external anal sphincter that was left intact. Courtesy of Sigmar Stelzner and Thilo Wedel, Institute of Anatomy, University of Kiel. With permission of Institute of Anatomy, University of Kiel, Germany

A 60-year-old male patient was referred to our hospital with a high-grade differentiated rectal adenocarcinoma. The pelvic MRI revealed a tumor at the lower rectum (its lower border was 1.5 cm from the anal verge) that invaded puborectalis muscle to a length of 9 mm on the right side. Moreover, the CT scan proved the absence of any distant metastasis. Given the tumor location and the absence of distant metastases, the patient went through manometric evaluation of anorectal function and clinical assessment with the Wexner scale score for incontinence (Table 1) before the beginning of neoadjuvant therapy in order to determine whether preservation of anal sphincteric complex could be a choice. After the completion of neoadjuvant treatment, patient was reassessed with pelvic MRI in which good response of tumor was observed. Due to the comprehensive sphincter function (pre-op Wexner score, 0), it was decided to perform a new sphincter-preserving technique without compromising the oncologic result. The patient was placed in the Lloyd-Davis position. The operation included an abdominal and a perineal phase with a total length of 5 h (skin to skin). For the abdominal phase, a midline incision from a point about 4 cm below the xiphoid to the pubis was performed in order to allow unrestricted view of the large intestine. Following Todds’ avascular plane the sigmoid was mobilized and the descending colon and splenic flexure afterwards. After the dissection of the inferior mesenteric artery and vein, they were ligated with a high tie. The following step was the dissection of the rectum as guided by the embryological planes for total mesorectal excision (TME) [7]. The dissection extended to the pelvic floor consisting of the levator ani muscle (LAM). Pelvic floor dissection at the tumor’s side was extended up to LAM’s attachment to the internal obturator fascia, while in the contralateral side it was directed to expose puborectalis muscle close to anorectal junction. The perineal phase started with a right hemi-circular incision at the level of intersphincteric line. The dissection plane followed the intersphincteric space to take out the right half of internal anal sphincter (IAS) and then moved to the right ischioanal fossa to include the deep part of ipsilateral external anal sphincter (EAS) in the specimen. The macroscopic margin from the tumor was 10 mm. Attachment of right LAM at internal obturator fascia was cut and dissection plane integrated entering the pelvic cavity. At the left side, the hemicircular line completed in an eccentric way to preserve the main mass of the left half of IAS and to leave intact the EAS. The lateral anal canal wall was transected at the upper edge of anal columns, while entering the pelvic cavity was performed by cutting the attachment of puborectalis muscle at the lateral rectal wall. A graphic representation of the surgical planes is presented in Figs. 1 and 2. A transection of the proximal colon was achieved with a stapler device. Bowel continuity was achieved with hand-sewn colo-anal anastomosis which was protected by a diverting loop ileostomy. Total blood loss was 1.5 units (750 ml).

**Table 1:** Characteristics of patient’s anorectal function

Characteristic	Pre-operative	Post-operative	Normal values (males)
Mean maximum anal resting pressure (mmHg)	68	50	59–74
Instant maximum squeeze anal pressure (mmHg)	175	110	60–220
Prolonged maximum squeeze anal pressure (mmHg)	120	45	40–200
Anal sphincter length (cm)	3.8	2.4	2.5–5
Minimum rectal volume for sustained anal relaxation (ml)	40	20	30–60
Rectal volume for first sensation (ml)	30	30	20–110
Rectal volume for permanent urge to defecate (ml)	150	60	60–170
Maximum tolerable rectal volume (ml)	220	100	110–320
Wexner score	0	7	0, perfect continence 20, major incontinence

## Results

**Fig. 3:**
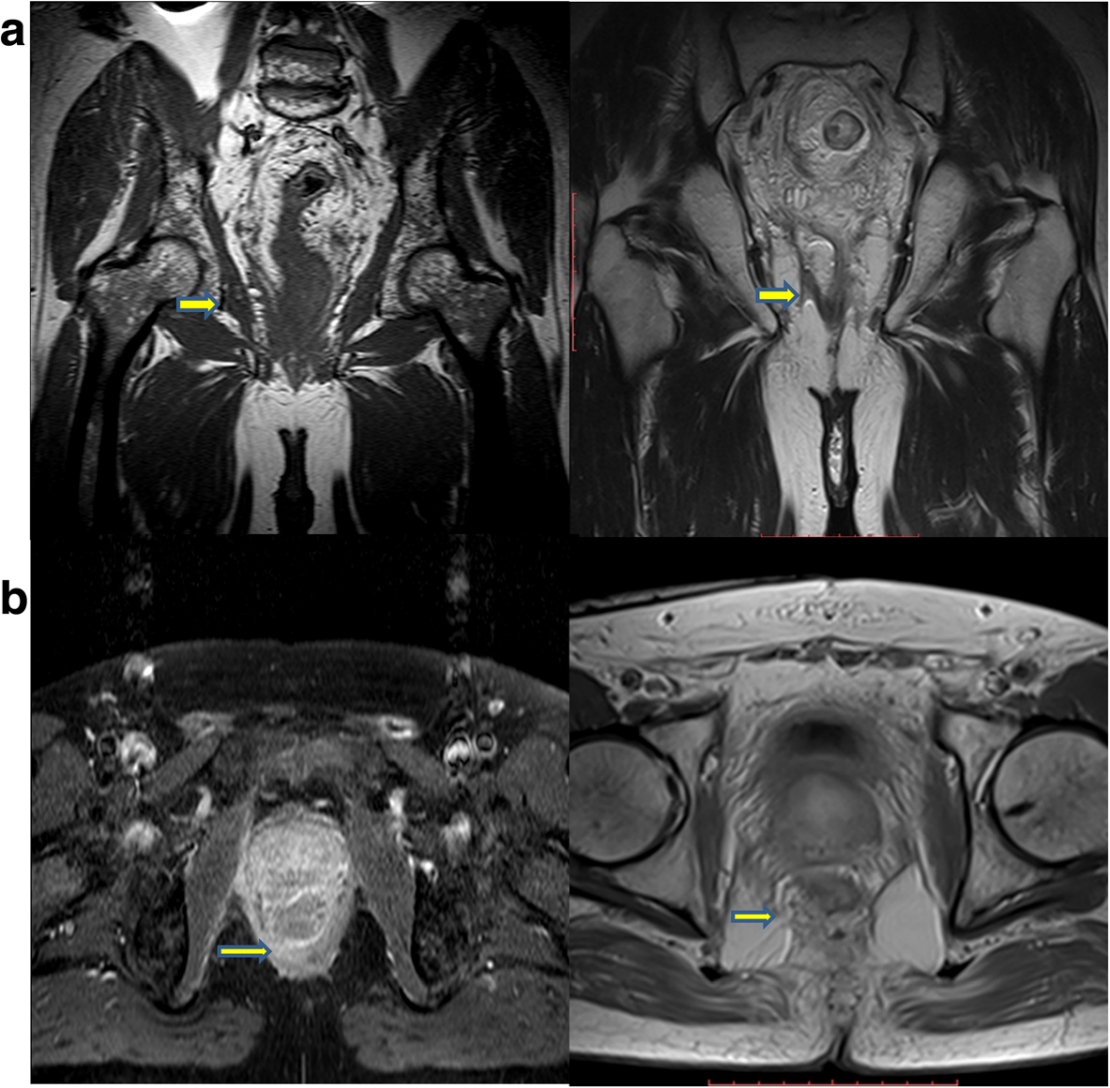
**a** MRI coronal view of patient pelvis pre- and post operatively (left and right, respectively) (arrow shows the infiltration of right portion of levetor ani muscle by the tumor and the defect remaining after the partial excision of levator muscle ani). Preoperative MRI was performed after neoadjuvant treatment (seventh week). **b** MRI axial view of the same patient’s area of interest

Postoperative route was uneventful with a total hospital stay of 6 days. Pathology proved the oncologic adequacy of the resection resulting to ypT3NxM0. According to the pathology report, no lymph nodes were harvested from the mesorectum (probably as a result of neoadjuvant therapy). MRI at the fourth postoperative week showed clearly the right aspect of anorectal junction free of tumor and the absence of ipsilateral LAM (Fig. 3a, b). The protective ileostomy was taken down 8 weeks after the surgery with no complications. The patient stayed in the hospital for 2 days and then was discharged. One month after restoring large bowel continuity, anorectal sphincter continence was re-evaluated by anorectal manometry and clinical assessment by Wexner score. A fair anorectal function was revealed which is in accordance with the findings of clinical assessment (post-op Wexner score, 7) (Table 1).

## Discussion

In the earlier days of colorectal surgery for malignant tumors of the lower third of rectum, the operation of choice was the abdomino-perineal resection (APR) in which the sigmoid, the rectum, and the anus were excised leaving the levator ani muscle complex intact in both sides. In this way, the specimen resembles an hourglass due to the characteristic “waist” in the middle [8]. However, given the incomplete resection rate and the high local recurrence compared to low anterior resection of rectum (LARR) [9] colorectal surgical community has nowadays moved towards the ELAPE. The last one has proved to be superior in oncologic terms compared to conventional APR. Its superiority relies largely on the fact that apart from the sigmoid, rectum, and anus, the levator ani complex is removed as well, providing a cylindrical (waist-free) specimen, reducing by this mean, tumor involvement at circumferential resection margin. As with APR, the patient ends up with a permanent colostomy. In fact, this is the main disadvantage of both operations affecting patients’ quality of life. In an attempt to reduce the frequency of permanent colostomy in low rectal cancer surgery a better selection of patients has been suggested. So, in cases with very low rectal lesions, no involvement of the external anal sphincter or the levator ani muscle complex, and adequate preoperative sphincter function and continence, ISR is preferred as it preserves anal sphincteric function to some extent. This is achieved by entering the intersphicteric space and dissecting the internal from the external anal sphincters, leaving the later almost intact [4]. Attempts for function-preserving procedures with partial external anal sphincter resection have been described in cases with external anal sphincter infiltration [10]. Moreover, in a special sub-group with unilateral puborectalis muscle infiltration and adequate sphincteric function, HLE was proposed as an attempt to keep anorectal function and achieve oncologic adequacy. A comprehensive summary of the current surgical procedures for low rectal cancer is presented in Table 2. Noh et al. proved that robotic/laparoscopic HLE yield oncologic results comparable to those of a standard ELAPE, while offering the patient the unparalleled advantage of avoiding a permanent colostomy [11]. According to them, an open approach is not feasible since the surgeon lacks of a clear view of the surgical field. Since the open approach still remains the standard of care in rectal cancer surgery, we tried to perform HLE by this way. Herein, we show that an open approach not only is feasible but also can potentially be served as a promising alternative for laparoscopic or robotic HLE since the latter two forms are not widely popularized among the surgical community yet. Moreover, being able to perform the open approach is important even among those surgeons who are trained on the laparoscopic and/or robotic techniques because knowing this alternative would allow them to overcome difficulties that would require the conversion of the surgery (from laparoscopic to an open one) with minimum oncologic cost for the patient. However, laparoscopic and robotic procedures overcome the open one concerning the enhanced vision and appreciation of the field [12, 13]. It is reasonable some concerns to be raised regarding the oncologic radicality since anatomic borders among LAM, PRM and the deep part of the EAS are not very clear [14]. Indeed, the heated debate regarding the anatomy of anal canal dates back to 1897. At that time, it was identified that some muscle fibers of the “pubococcygeus,” instead of inserting into the coccyx, loop around the rectum, continue on to the opposite side and thus form a different muscle, the PRM. Since then, the EAS is perceived as a three-part structure with the PRM being part of the LAM; PRM is located just below the LAM and EAS extends down. The very close relation of the deep part of the EAS and PRM has led some authors to consider them as one muscle [15, 16]. Baring this debate in mind, in order to enhance the oncologic safety of the procedure, the deep part of ipsilateral to tumor EAS is included in the surgical specimen. Moreover, a macroscopic margin 10 mm of the transection line from the lower border of tumor ensures the oncologic adequacy further. The oncologic value of the open approach seems to be equal to that of the other approaches, as proved by the pathology of the specimen and the MRI at the fourth post-operative week that shows clearly the right aspect of anorectal junction free of tumor and the absence of ipsilateral LAM (Fig. 2a, b). The major advantage of the open procedure is the maintenance of continence, as proved by the postoperative clinical assessment of patient after restoring large bowel continuity (post-op Wexner score, 7) and the anorectal manometry findings (which in our case, revealed a fair anorectal function). In fact, the efficiency of the operated sphincter is acceptable since only a part of the deep portion of the EAS is removed. Preservation of internal anal sphincter at the contralateral to tumor side might also add to the whole sphincteric function and particularly at rest and during the sleep.

**Table 2:** Summary of the current trends in surgical procedures for low rectal cancers

Operation	Technical description	Indication	Disadvantages	Reference
Abdomino perineal resection (APR)	Sigmoid, rectum, and anus are excised sparing the levator ani muscles complex (hourglass-like specimen)	Lesions at the lower third of the rectum	Poor oncologic outcome, permanent colostomy	Hussain et al. [8]
Extralevator abdomino-perineal excision (ELAPE)	APR + excision of the levator ani muscles complex (cylindrical specimen)	Lesions at the lower third of the rectum	Permanent colostomy	Carpelan et al. [17]
Intersphicteric resection (ISR)	Surgical plane in the intersphicteric space, dissection of the internal anal sphincter, saving the external sphincter	• Lesions at the lower third of the rectum that do not involve the levator ani muscles • Good pre-operative sphincter function and continence	May not be suitable for patients that have undergone neoadjuvant treatment	Schiessel et al. [4]
Subtotal intersphincteric resection/partial external sphincteric resection	ISR + partial external anal sphincter resection	• Lesions of the lower third of the rectum invading part of the external anal sphincter • Good pre-operative sphincter function and continence	Not applicable for lesions invading the levator ani muscle	Mukai et al. [10]
Hemilevator excision (HLE)	Resection of the levator ani muscle, the deep part of external anal sphincter and the internal sphincter ipsilaterally. The contralateral ones are preserved	• Lesions at the lower third of the rectum involving the levator ani muscle in one side • Good pre-operative sphincter function and continence	Not applicable for cancers circumferentially infiltrating levator ani complex	Noh et al. [11]

## Conclusions

This is the first attempt at Greece to perform a technique which targets the saving of anal sphincter for very low rectal cancers with extension to the puborectalis muscle. This is the first procedure with removal of puborectalis muscle and partial excision of external sphincter with preservation of anal function. This innovative procedure requires full knowledge of pelvic anatomy. The surgical team must have experience to the standard TME. This procedure is the hope for a life without colostomy for patients with these tumors. Undoubtedly, a larger number of cases is demanded to draw firm conclusions since we have to take into account that anatomic characteristics such as gender, body mass index, etc. might affect the feasibility of the procedure.

## Data Availability

The data that support the findings of this study are available from John Tsiaoussis, but restrictions apply to the availability of these data, which were used under license for the current study, and so are not publicly available. Data are however available from the authors upon reasonable request and with the permission of John Tsiaoussis.

## Abbreviations

APE: Abdomino-perineal excision
CRM: Circumferential resection margins
EAS: External anal sphincter
ELAPE: Extralevator abdomino-perineal excision
HLE: Hemilevator excision
IAS: Internal anal sphincter
ISR: Intersphicteric resection
LAM: Levator ani muscle
LAR: Low anterior resection
PRM: Puborectalis muscle
TME: Total mesorectal excision
